# Constitutive Androstane Receptor: A Peripheral and a Neurovascular Stress or Environmental Sensor

**DOI:** 10.3390/cells9112426

**Published:** 2020-11-06

**Authors:** Fabiana Oliviero, Céline Lukowicz, Badreddine Boussadia, Isabel Forner-Piquer, Jean-Marc Pascussi, Nicola Marchi, Laila Mselli-Lakhal

**Affiliations:** 1 Toxalim (Research Centre in Food Toxicology), Université de Toulouse, INRAE, ENVT, INP-Purpan, UPS, 31027 Toulouse, France; fabiana.oliviero@inrae.fr (F.O.); celine.lukowicz@unil.ch (C.L.); 2 Cerebrovascular and Glia Research, Institute of Functional Genomics (UMR 5203 CNRS–U 1191 INSERM, University of Montpellier), 34094 Montpellier, France; badreddine.boussadia@gmail.com (B.B.); Isabel.Forner-Piquer@igf.cnrs.fr (I.F.-P.); jean-marc.pascussi@inserm.fr (J.-M.P.)

**Keywords:** constitutive androstane receptor, liver, brain, neurovascular unit, blood-brain barrier, stress sensor, environmental contaminants

## Abstract

Xenobiotic nuclear receptors (NR) are intracellular players involved in an increasing number of physiological processes. Examined and characterized in peripheral organs where they govern metabolic, transport and detoxification mechanisms, accumulating data suggest a functional expression of specific NR at the neurovascular unit (NVU). Here, we focus on the Constitutive Androstane Receptor (CAR), expressed in detoxifying organs such as the liver, intestines and kidneys. By direct and indirect activation, CAR is implicated in hepatic detoxification of xenobiotics, environmental contaminants, and endogenous molecules (bilirubin, bile acids). Importantly, CAR participates in physiological stress adaptation responses, hormonal and energy homeostasis due to glucose and lipid sensing. We next analyze the emerging evidence supporting a role of CAR in NVU cells including the blood–brain barrier (BBB), a key vascular interface regulating communications between the brain and the periphery. We address the emerging concept of how CAR may regulate specific P450 cytochromes at the NVU and the associated relevance to brain diseases. A clear understanding of how CAR engages during pathological conditions could enable new mechanistic, and perhaps pharmacological, entry-points within a peripheral–brain axis.

## 1. Introduction: CAR Governs Detoxification Mechanisms 

The Constitutive Androstane Receptor (CAR), a key nuclear receptor (subfamily 1, group I, member 3 (NR1i3)), displays a prominent functional expression in peripheral organs and an emerging role in the brain. Here, we examine CAR as an element responding to environmental or stress challenges, preserving cellular and multi-organ homeostasis. CAR was originally defined as a xenobiotic nuclear receptor that controls the hepatic detoxification of foreign chemicals and endogenous bile acids [1]. CAR is the mediator of phenobarbital-induced cytochrome P450 enzymes expression in the liver [2,3]. CAR directly regulates the expression of an array of phase I and II xenobiotic metabolism enzymes and multi-drug transporters (Table 1) [4,5]. Specifically, CAR controls the inductive expression of the CYP phase I enzymes CYP2B, CYP3A, CYP2C, contributing to the detoxification of numerous drugs and environmental chemicals. Furthermore, CAR activation results in the upregulation of phase II enzymes such as uridine diphosphate glucuronosyltransferase (UGT), sulphotransferases (SULT), and efflux and uptake transporters such as multidrug resistance mutation 1 (MDR1), multidrug resistance proteins (MRPS), and organic-anion-transporting polypeptides (OATP) [6,7]. Via these enzymes and transporters, CAR governs the detoxification of endogenous bile acids and bilirubin, which can cause hepato-toxicity if accumulated [8,9]. This evidence outlines CAR as a player regulating key metabolic and transporter machineries involved in a myriad of endogenous and protective cellular processes, applicable to peripheral organs and the brain.

Importantly, microarray analyses identified additional sets of CAR target genes involved in hepatocyte proliferation, glucose and lipid metabolism [10,11]. The latter was confirmed in functional studies revealing that selective activation of CAR alleviates high fat diet-induced obesity and type 2 diabetes [12,13]. Furthermore, CAR is activated by cellular stress as induced by fasting, caloric restriction [14,15] or hypoxia [16]. CAR regulatory pathways include AMP-activated protein kinase (AMPK) [17], a key player maintaining intracellular homoeostasis, and stress activated protein kinase (SAPK) [18]. Based on this evidence, we here review how CAR, through key peripheral and central functions, acts as a stress sensor engaging and responding to toxic, environmental, or metabolic insults. 

## 2. CAR Has a Particular Mechanism of Action: Direct and Indirect Activation

**Figure 1:**
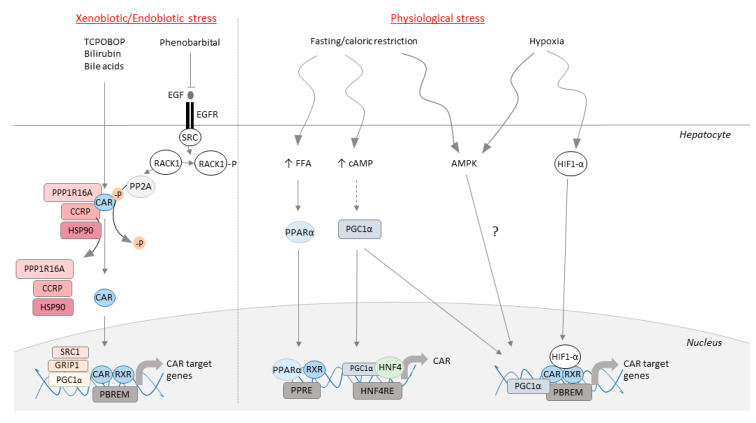
CAR is a sensor for xenobiotic/endobiotic and physiological stress. CAR is maintained in the cytoplasm by a complex of chaperone proteins (PP1R16A, CCRP, HSP90) and can be activated through direct or indirect processes by a xenobiotic or endogenous molecule. Dephosphorylation of CAR allows it to be free from its chaperone complex, to migrate to the nucleus, heterodimerize with RXR, recruit co-activator factors and allow transcription of its target genes. Gene expression of CAR is induced in response to fasting and caloric restriction through nuclear receptors PPARa and HNF4. Hypoxia induces nuclear translocation of CAR and expression of its target genes through HIF1.

The crystal structure of CAR, published in 2004 [19,20], outlined the sites responsible for its constitutive activity. CAR contains a single-turn helix X located before the C-terminal AF2 helix that favors an active conformation of the receptor. The intrinsic constitutive nature of CAR necessitates specific mechanisms of regulation aside from ligand binding. Specifically, CAR is sequestered in the cytoplasm in a phosphorylated active conformation, forming a complex with chaperone proteins: Cytoplasmic CAR retaining protein (CCRP), Heat shock protein (HSP90) and PPP1R16A (the membrane subunit of protein phosphatase 1β) [21,22]. TCPOBOP (murine form, 1,4-Bis-[2-(3,5-dichloropyridyloxy)]benzene) or CITCO (human form, 6-(4-Chlorophenyl)imidazo[2 ,1-b][1,3]thiazole-5-carbaldehyde O-(3,4-dichlorobenzyl)oxime) are synthetic CAR agonist interacting with the ligand-binding pocket of CAR to induce its nuclear translocation (Figure 1) [23]. This translocation requires the recruitment of phosphatase protein A2 (PP2A) responsible for dephosphorylating the threonine 38 (human form) or 48 (murine form) [24], thus releasing CAR from its cytosolic complex [21,22].

CAR can be activated by endogenous and exogenous molecules without direct binding. Phenobarbital induces CAR translocation indirectly by competing with the epidermal growth factor (EGF) for binding on its receptor EGFR. This prevents activity of Src kinase and dephosphorylates Receptor of activated C kinase 1 (RACK1). RACK1 activation of PP2A leads to dephosphorylation and nuclear translocation of CAR (Figure 1) [24]. Once in the nucleus, CAR heterodimerizes with its partner Retinoid X Receptor (RXR), and binds to its response elements: Phenobarbital response element module (PBREM) [25]. PBREMs are located on the promoters of CAR-target genes such as *CYP2B6* for the human form of CAR, or *Cyp2b10* for murine CAR. The CAR/RXR heterodimer recruits specific co-activators allowing its interaction with the transcription machinery. Glucocorticoid receptor interacting protein-1 (GRIP-1), Proliferator activated receptor coactivator 1α (PGC1α), and Steroid receptor co-activator (SRC-1) allow the transcription of CAR-target genes [26,27,28].

## 3. Functional Roles of CAR in Peripheral Organs

CAR is expressed mainly in the liver, and also in the intestines and kidneys [29]. Most of the functions of CAR as a stress sensor occur in the liver, the main peripheral organ responsible for xenobiotics and metabolic stress responses [30]. The generation of CAR deficient mice represented a milestone to understand the complex roles of CAR in physiological and pathological settings [31]. CAR deletion induces sensitivity to toxins due to the disruption of detoxification enzymes regulation. Thus, CAR coordinates the expression of hepatic genes involved in xenobiotic catabolism, including phase I and II biotransformation enzymes and transporters [32]. Importantly, two main groups of xenobiotics are described as modulators of CAR activity: drugs and environmental pollutants (Table 2 and Table 3). Hepatoprotection of CAR against xenobiotics does not only consist of induction of detoxification genes but also in specific gene repression. For instance, CAR prevents the induction of CYP4A, a major lipid peroxidation enzyme, by augmenting superoxide dismutase-3 (SOD) to limit oxidative stress [10].

### 3.1. CAR as an Endobiotic Stress Sensor

CAR controls the metabolism of endogenous molecules. The degradation product of heme, bilirubin, is a potentially highly neurotoxic endogenous compound in case of extended accumulation. Glucuronidation by UGT1A1 enzyme is the main detoxification pathway of bilirubin, then secreted into the bile by MRP2 active transporter. CAR is involved in bilirubin clearance by inducing UGT1A1 and Glutathione S-transferase A1 (GSTa1) enzymes [77], as well as OATP2 and MRP2 transporters [78].

CAR activation in mice induces the expression of enzymes (CYP3A11, SULT2A1) and transporters (MRP3) involved in bile acid metabolism and elimination [56]. Bile acids, produced by the liver, are necessary for cholesterol elimination and dietary lipids absorption. Hepatic cholesterol is degraded in two primary bile acids, cholic acid (CA) and chenodeoxycholic acid (CDCA). In the intestine, these bile acids are hydroxylated by the microbiota into secondary bile acids, deoxycholic acid and lithocholic acid (LCA), reabsorbed and transported to the liver [79]. Wild type mice subjected to a diet containing 0.5% of LCA present Cyp2b10 induction which is not seen in CAR knock-out mice subjected to the same diet [56]. Furthermore, TCPOBOP-induced CAR activation protects the liver from cholestasis by allowing the production of non-toxic bile acids [80]. CAR seems to act independently from Farnesoid X Receptor (FXR) receptor, a known xenosensor of bile acids. In FXR and PXR deficient mice, elevated cholic acid levels induce expression of CAR and its target genes which metabolize potentially-toxic cholic acid [78].

### 3.2. CAR as a Regulator of Steroid and Thyroid Hormones

The involvement of CAR in hormone regulation was first suggested when androstanol and androstenol were identified as CAR ligands. These steroids inhibit CAR activity by inducing the dissociation of CAR from the co-activator SRC1 [2]. Progesterone and testosterone also inhibit CAR activity [59]. Steroid hormone levels are maintained by a dynamic balance between synthesis and inactivation due to limited storage capacity. Therefore, coordination between synthesis and biotransformation is necessary to maintain normal physiological functions. Many CYPs (CYP11, CYP17, CYP19, CYP21) are responsible for steroid hormone synthesis, catabolism and inactivation [81]. The liver is the main site of steroid catabolism where CAR plays an important role through regulation of CYPs and sulfotransferases expression. TCPOBOP activation of CAR induces estrogen catabolism to promote its excretion [82]. CAR also regulates estrogen sulfotransferase (EST) encoded by the *SULT1E1* gene which catalyzes the conjugation of a sulfate on estrogens, producing inactive forms [83].

Several studies outline a link between CAR and thyroid hormones activity. Phenobarbital chronic treatment leads to thyroid hypertrophy in rats and humans [84]. Other studies revealed that phenobarbital or phenytoin activation of CAR lowered circulating thyroxine (T4) levels [15,84,85]. CAR is involved in thyroid hormone catabolism through regulation of phase II enzymes UGT1A1 and SULT1A1 [4,86]. Experimental evidence demonstrated CAR regulation of the catabolism of the stress hormone corticosterone [87]. CAR knockout mice developed hypercorticism associated with obesity, glucose intolerance, insulin insensitivity, dyslipidemia and hepatic steatosis [87]. Remarkably, the latter modifications were absent, or minor, in CAR knockout females, developing similar metabolic disorders only when ovariectomized. Analysis of the hepatic transcriptome revealed a role of CAR in the catabolism of corticosterone [87]. CAR deletion resulted in down-regulation of enzymes involved in the hepatic catabolism of steroid hormones, specifically Hydroxy-Delta-5-Steroid Dehydrogenase, 3 Beta- And Steroid Delta-Isomerase 1 (Hsd3b1), Hydroxy-Delta-5-Steroid Dehydrogenase, 3 Beta- And Steroid Delta-Isomerase 5 (Hsd3b5), Hydroxy-Delta-5-Steroid Dehydrogenase, 11 Beta- And Steroid Delta-Isomerase 1 (Hsd11b1), Aldo-Keto Reductase Family 1 Member C14 (Akr1c14), Steroid 5 Alpha-Reductase 1 (Srd5a1) and Aldehyde Dehydrogenase 3 Family Member A2 (Aldh3a2) [87]. Altogether, these results highlight the important role of CAR in the maintenance of endocrine and metabolic equilibrium. The CAR-dependent regulation of hormone catabolism constitutes a lever for the maintenance of energy homeostasis.

### 3.3. CAR as a Sensor of Fasting and Caloric Restriction

The activity of CAR is modulated according to physiological and pathophysiological conditions (Figure 1). Resistance to weight-loss during extended fasting or caloric restriction requires the establishment of specific metabolic pathways. CAR deficient mice present a defect in extended-fasting resistance to weight loss [15]. Importantly, fasting activates CAR through interaction of PGC1α and Hepatocyte nuclear factor 4 α (HNF4α) with response elements that regulate expression of CAR [14]. Nuclear receptor Peroxisome proliferator-activated receptors α (PPARα) is essential in CAR induction during response to fasting [88]. The glucocorticoid receptor (GR) is also involved, as its response elements have been identified on the CAR promoter [89]. Finally, a study conducted on HepG2 cells revealed the involvement of SAPK and ETS Like-1 (Elk1) in regulating CAR expression [18]. Collectively, these data support CAR as a nuclear receptor that reacts to nutritional conditions such as fasting and caloric restriction.

### 3.4. CAR as a Glucose Sensor

A role of CAR in glucose homeostasis was first hypothesized as a result of clinical observations obtained from phenobarbital-treated diabetic patients who presented improved insulin sensitivity and decreased glycaemia levels [90,91]. Experimentally, diabetic mice present improved glucose tolerance following treatment with the CAR agonist TCPOBOP [12]. Improvement of glucose tolerance is mainly due to the suppression of hepatic glucose production. CAR activation represses the gluconeogenesis limiting enzymes Phosphoenolpyruvate carboxykinase (PEPCK) and Glucose 6-phosphatase (G6Pase). A number of mechanisms were suggested, including competition of CAR with Forkhead box protein O1 (FoxO1) and HNF4α for binding on Iron-responsive element (IRE) and Hepatocyte nuclear factor 4 α responsive element (HNF4RE), respectively located on promoters of PEPCK and G6Pase [92]. Furthermore, CAR could bind to SRC2/GRIP1 and PGC1α which are two co-activators of HNF4α, lowering expression of gluconeogenesis genes [93]. Additional evidence suggests that nuclear translocation of CAR allows physical interaction with PGC1α, allowing the recruitment of E3 Culline Ligase and interaction to Promyelocytic leukemia (PML) nuclear bodies. This leads to PGC1α degradation by the proteasome and consequent repression of gluconeogenesis genes [94]. Finally, data suggest the action of CAR through SULT2b1 regulation on HNF4α deacetylation, which could prevent CAR nuclear translocation and action on gluconeogenesis genes [95]. CAR repression of gluconeogenesis genes was confirmed on human hepatocyte primary cultures [96]. Overall, this reveals a glucose sensing action of CAR through regulation of hepatic gluconeogenesis.

### 3.5. CAR as a Lipid Sensor

The role of CAR in the regulation of lipid metabolism remains controversial, with studies reporting both anti-lipogenic [12,13] and pro-lipogenic functions [97,98]. Chronic treatment with CAR activators, such as phenobarbital or valproic acid, is associated with hepatic metabolic disorders [99,100]. These clinical observations were confirmed using animal models, revealing a CAR-dependent control of fatty acid catabolism and hepatic lipogenic genes. CAR acts as an anti-lipogenic factor by interfering with PPARα on β-oxydation of fatty acids [101]. In ob/ob obese mice subjected to a high fat diet, CAR activation leads to a decreased hepatic steatosis by inhibiting de novo lipogenesis via Stearoyl-CoA desaturase-1 (Scd1), Fatty acid synthase (Fas), Acetyl coa carboxylase (Acc), and SREBP-1c repression [12,13]. Furthermore, CAR may act on de novo lipogenesis genes through the LXR nuclear receptor, regulator of hepatic lipogenesis genes. CAR could contribute to the inactivation of oxysterols which are endogenous ligands of LXR through regulation of Sult2B1b sulfotransferase expression. Oxysterol inactivation leads to decreased LXR activity and reduction of the LXR–SREBP pathway [102]. Accordingly, Sult2B1b deficient mice treated with TCPOBOP do not present repression of de novo lipogenesis genes [12].

However, recent data reported a prolipogenic effect of CAR, inducing hepatic lipid accumulation upon its activation [98]. This occurs through the induction of hepatic lipogenic genes, including patatin-like phospholipase domain-containing protein 3 (*Pnpla3*), a gene whose polymorphism is associated with the pathogenesis of non-alcoholic fatty liver diseases (NAFLD). The underlying mechanism involves the transcription factor Carbohydrate-responsive element-binding protein (ChREBP), a master regulator of hepatic carbohydrate-lipid metabolism [98]. The same prolipogenic effect was reported on human hepatocytes primary cultures, revealing that Thyroid hormone responsive protein (*Spot14*), a CAR target gene, is an important modulator of hepatic lipogenesis [97].

The contradictory effect of CAR on lipid metabolism may be explained considering the dissimilar physiological settings across studies. CAR appears to inhibit lipogenesis in a situation of metabolic stress induced by a high-fat diet, while activating it when a chemical stress is caused by the presence of a pharmacological agonist. Lipid droplet accumulation following activation of CAR by a xenobiotic could allow the neutralization of the xenobiotic before elimination from the cell. This hypothesis remains to be verified. Overall, these data indicate CAR as a stress sensor, responding in a stress-depend manner to allow cell homeostasis.

### 3.6. CAR as a Hypoxia Sensor

CAR was reported to cross-talk with Hypoxia inductible factor (HIF1) [16]. Hypoxia is a pathologic condition that activates HIF1 transcription and AMPK in the same way as energetic depletion and oxidative stress [103]. HIF1 is degraded during normoxic conditions and it is stabilized by hypoxia to regulate transcription of its target genes: Vascular Endothelial Growth Factor (*VEGF*), erythropoietine (*EPO*) and glycolytic enzymes. Interestingly, treatment of mice with a HIF1 activator induces nuclear translocation of CAR and the expression of Cyp2b10. Furthermore, CAR can interact with HIF1 when binding on PBREMs [16]. This initial evidence suggests the engagement of CAR during reduced oxygen levels as an attempt to maintain cell homeostasis.

### 3.7. CAR Intestinal Response to Inflammatory Stress

CAR is down-regulated in intestinal biopsies obtained from Crohn’s disease or Ulcerative Colitis patients and in colitis mouse tissues [104]. Consistently, CAR activation accelerates intestinal mucosal healing both in vitro and in vivo, suggesting that CAR plays a role in the maintenance of intestinal mucosal integrity, while CAR dysfunction could contribute to the pathogenesis of inflammatory bowel diseases. In addition, a link between CAR and the gut microbiota was reported. In mice, pharmacological activation of CAR by its ligand TCPOBOP impacted the microbiome composition and down-regulated bile-acid-metabolizing bacteria in the intestine [105]. A deficiency of CAR also modified the microbiota, increasing the pro-inflammatory bacteria and cytokines [106]. CAR may act on immune surveillance to prevent the colonization of harmful bacteria [106]. These data suggest that CAR regulates microbiota composition and responds to intestinal inflammatory stresses.

### 3.8. CAR Protects from Acute Kidney Injury

A role of CAR was recently demonstrated in mediating a kidney-liver cross-talk in Acute kidney injury (AKI) which is characterized by the sudden impairment of kidney function [107]. CAR activation by its agonist prevented the development of AKI-induced fatty liver and liver injury, and improved kidney function [107]. The protective effect of CAR agonist was abolished in CAR knockout mice. These results suggest that CAR could be a target in the management of hepatic steatosis and kidney function in patients with AKI.

## 4. Functional Roles of CAR in the Brain: Focus on the Neurovascular Unit

The brain integrates multiple inputs from and to the periphery, providing the adequate adaptive response to environmental changes. This includes the regulation of energy homeostasis based on highly coordinated interactions between the brain and peripheral metabolic organs [108]. Here, we examine the available evidence indicating expression of CAR in the brain, then extending to its potential role in a peripheral–brain interplay.

### 4.1. Brain Expression, Regulation and Function of CAR

While studied in peripheral organs, it is only recently that the functional expression of CAR was examined in the brain, in healthy and pathological conditions. Outing its expression patterns at the neurovascular unit (NVU) is important to unveil novel functional aspects at a key physiological brain–peripheral interface and within a multi-cellular neuronal, glial, and cerebrovascular structure [109,110,111]. Within the NVU, the blood–brain barrier (BBB) represents a key dynamic interface functioning as a protective brain gatekeeper [109,111] and as a hindrance for the delivery of systemically administered brain xenobiotics [112].

Early reports described mRNA and protein levels expression of CAR in the brain. In humans, analysis of mRNA in whole tissue homogenates obtained from one adult brain specimen showed detectable, although low as compared to liver and intestine, expression of CAR [113]. Others reported CAR mRNA in brain areas, specifically in the nucleus accumbens, caudate nucleus, and putamen [114,115]. In rodents, the expression of CAR was demonstrated at the mRNA or protein levels in the cerebral cortex, hippocampus, midbrain and the cerebellum [116,117,118,119]. An available human protein ATLAS dataset outlines the expression of CAR in the brain, further indicating levels in the cerebral cortex, hippocampus, amygdala, hypothalamus and the basal ganglia [120]. To date, no specific studies have examined the impact of gender and aging on brain CAR expression.

Supporting a possible role at the cerebrovascular interface, CAR mRNA and protein expression were shown in an in vitro BBB model, using human-derived cerebral endothelial cells [121]. The functional expression of CAR, and other cognate nuclear receptors, at the BBB was demonstrated by quantifying specific downstream P450 cytochromes or MDR gene targets [19,122,123,124,125,126]. Interestingly, acetaminophen treatment in mice increased the functional expression of Abcb1 transporter (P-gp) at the BBB by a CAR-dependent mechanism [126]. These results are significant, considering the strategic expression of xenobiotic nuclear receptors at the NVU interface. In other brain cell types, the role of CAR was examined in the settings of chemotherapy and pesticide-induced neurotoxicity [127]. In neuroblastoma (SH-SY5Y) and glioblastoma (U373-MG) cell lines, up-regulation of CAR mRNA and specific P450 cytochromes (CYP3A4, CYP2C8, etc.) was reported following treatment with cyclophosphamide, also increasing reactive oxygen species (ROS) production, and upregulating the expression of pro-apoptotic markers caspase-3, caspase-9, Bax, and p53 [127].

Modulation of CAR directly impacts the expression of biotransformation transporters and enzymes, possibly affording neuroprotection [7,124,128,129,130]. Available evidence supports a use for CITCO, a CAR agonist, as a potential therapy for gliomas. CITCO inhibits the growth and expansion of cancer stem cells by inducing cell cycle arrest and apoptosis, without affecting primary astrocytes [129]. Additional evidence indicated that stimulation of primary cultures of porcine brain capillary endothelial cells with CITCO provoked a significant up-regulation of Abcb1 (P-glycoprotein) and Abcg2 (breast cancer resistance protein) efflux-transporters at the RNA, protein and transport levels [131]. Another study showed that exposure to Triclocarban (3,4,4′-trichlorocarbanilide), an antibacterial, induced apoptosis of embryonic neuronal cells. The mechanism encompassed a caspase-3 dependent process with a CAR-mediated signaling activation. Furthermore, triclocarban induced CAR hypomethylation along with a disruption of the epigenetic status of neuronal cells and inhibiting post-translational protein modifications [118]. Collectively, this initial evidence supports the continuous investigation of the varying roles of CAR in brain cells.

### 4.2. CAR and Brain Disease Conditions: Initial Clinical and Experimental Clues

In humans, a genetic association study identified a mutation of CAR (NR1I3) in a cohort of pediatric subjects affected by the Kleefstra syndrome (KS) (OMIM#610253), a condition characterized by neurodevelopmental delay, dysmorphic features, behavioral and intellectual disabilities. However, the pathophysiology of CAR mutation in KS patients remains understudied [132]. From a pre-clinical stand point, loss of CAR in mice was associated with memory defects and anxiety-like behavior [133]. Electroencephalographic changes in CAR−/− mice during sleep or awake periods were found to correlate with memory outcome. This phenotype was accompanied by morphologic glial modifications suggestive of a mild neuro-inflammatory processes. Moreover, expression of the tight junction protein ZO-1 was reduced in isolated brain capillaries, pointing to BBB permeability [133]. Taken together, this initial indication suggests that pharmacological, or genetic, modulation of CAR could be one element perhaps contributing to neurological dysfunctions [118,133]. Understanding the functional relevance of CAR expression in brain cells may be significant to unravel new molecular mechanisms involved in neurodevelopmental diseases.

The memory defects and anxiety-like behavior displayed by the CAR knock-out mice could be linked with the endocrine and metabolic disorders reported in this model [87,133]. A similar link was suggested in another study, indicating that CAR selective activation alleviates high fat diet-induced obesity. The authors suggested that the hypothalamic and pituitary functions of CAR may have contributed to the hepatic phenotype [13]. Although CAR is not expressed in brown and white adipose tissues (BAT, WAT), the authors observed increased BAT energy expenditure, and activation of adipose triglyceride lipase gene expression in WAT upon CAR activation. This suggests an indirect effect of CAR activation in tissues outside the adipose tissue [108]. Tissue-specific CAR knockout mice (e.g, hepatic) could constitute a tool to further elucidate the varying roles of CAR in physiological and pathological settings.

## 5. Conclusions: Can We Integrate CAR within a Peripheral–Brain Axis?

**Figure 2:**
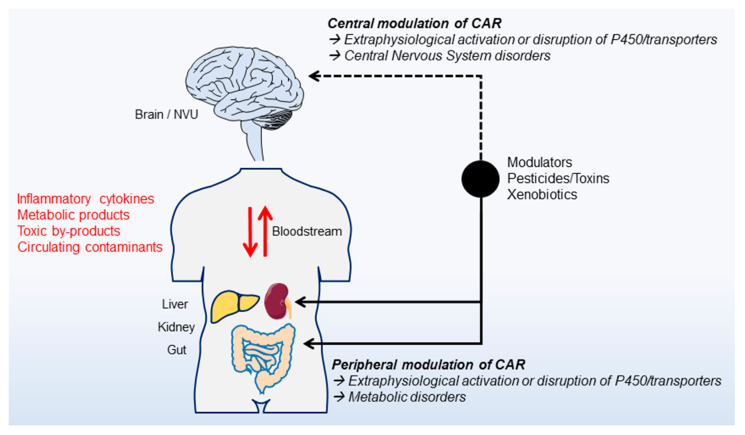
Modulation of CAR activity and a brain-peripheral dialogue. Peripheral or central activities of CAR can be modulated by endogenous or exogenous molecules (e.g., pesticides, toxins, xenobiotics), leading to a disruption of detoxifying p450 cytochromes and transporters regulation. A pathological brain-peripheral dialogue may enable disease conditions via soluble circulating blood factors, such as pro-inflammatory cytokines, metabolic or toxic by-products.

Existing evidence supports a multi-facet role of CAR, capturing the physiological state of varying cell types and contributing to organs homeostasis. Available data support the hypothesis that CAR functions may extend to a dialogue between multiple organs, perhaps including the central nervous system (Figure 2). The role of CAR could differ according to brain regions as suggested [134]. Furthermore, in the brain, CAR may be involved in the control of energy homeostasis through a cross-talk with the liver or the adipose tissue [13,87,133]. These associations are supported by clinical and experimental data revealing a link between obesity, cognitive impairment and BBB dysfunction. Rats fed with a high fat/sugar diet present with hippocampal BBB permeability, contingent to weight gain and concomitant to hippocampal-dependent learning defects [135]. Pathological conditions such as inflammatory bowel diseases (IBD; e.g., ulcerative colitis (UC); Crohn’s disease (CD)) [136,137] can present extra-intestinal, brain symptoms. Approximately 3% of subjects suffering from IBD display neurological symptoms [137] and cerebrovascular disorders occur in 0.12–4% of cases [138]. The latter is important as BBB breakdown is emerging as a participant mechanism of dysregulated peripheral–CNS interplay, promoting or contributing to brain diseases [110,139].

The exact role of nuclear receptors within the peripheral–brain axis needs to be fully deciphered. Experimentally, a CAR mediated microbiota–gut–brain communication was suggested [140] given the emerging role of this receptor in the brain [133] and in microbiota–gut interaction [104]. Experimentally, lack of CAR expression in mice was associated with metabolic disruptions including obesity, diabetes and hepatic steatosis [87] Concomitantly to BBB permeability, impairment in recognition memory and increased anxiety-like behavior were observed [133]. These studies support the hypothesis of a multi-organ pathological impact of CAR deletion. Further studies are required to understand whether the peripheral metabolic disorders lead to brain dysregulations or whether NVU cells damage in specific brain areas is the initiator of peripheral pathology. In summary, a holistic role of CAR fits within the accumulating evidence indicating a peripheral–brain interplay, as occurring in metabolic and CNS diseases. Modulating CAR during pathological conditions could represent a new strategy to prevent or target metabolic modifications impacting the periphery and the brain.

## Figures and Tables

**Table 1:** Constitutive Androstane Receptor (CAR) target genes involved in detoxification processes [4,5].

	Mice	Human
Phase I	*Cyp1a1*, *Cyp1a2*, *Cyp2a4*, *Cyp2b10*, *Cyp2c29*, *Cyp2c37*, *Cyp2c55*, *Cyp3a11*, *Nqo1*, *Aldh1a1*, *Aldh1a7*, *Akr1b7*, *Ces6*	*CYP1A1*, *CYP1A2*, *CYP2B6*, *CYP2C8*, *CYP2C9*, *CYP2C19*, *CYP3A4*, *CYP3A5*
Phase II	*Ugt1a1*, *Ugt1a9*, *Ugt2b34*, *Ugt2b35*, *Ugt2b36*, *Sult1e1*, *Sult2a1*, *Sult2a2*, *Sult3a1*, *Sult5a1*, *Gsta1*, *Gsta4*, *Gstm1*, *Gstm2*, *Gstm3*, *Gstm4*, *Gstp*, *Gstt1*	*UGT1A1*, *SULT2A1*
Transporters	*Mrp2, Mrp3*, *Mrp4*, *Oatp1a4*	*MDR1*, *PTGS2*, *GCK*, *PTPRN*, *ATP2B2*

**Table 2:** Environmental contaminants identified as CAR activators.

Contaminants	Species	References
Diphenamid (Pesticide)	Human	[33]
Phenothrin (Pesticide)	Human	[33]
Permethrin (Pesticide)	Rat	[34]
Perfluorocarboxylic acid, PFCA (Detergent)	Mice	[35,36]
Perfluorooctanoic acid, PFOA (Detergent)	Mice	[3,37]
Perfluorooctanesulfonic acid PFOS (Detergent)	Rat	[38]
Alachlor (Pesticide)	Mice	[39]
Arsenite (Chemical)	Mice	[39]
Azo dyes (Paint)	Mice/Rat	[40]
Bisphenol A (Chemical)	Mice	[39]
Butylate (Pesticide)	Mice	[39]
Chlorpropham (Pesticide)	Mice	[39]
Chlorpyrifos (Pesticide)	Mice	[39]
Cypermythrin (Pesticide)	Mice	[39]
Cyproconazole (Pesticide)	Mice	[41]
DBP, Di-n-butylphtalate (Plasticizer)	Rat	[42]
DDE, Dichlorodiphenyldichloroethylene (Pesticide)	Rat	[43]
Di-isononyl phthalate (DiBP) (Plasticizer)	Human	[44]
O,p-DDT,1,1,1-Tichloro-2-(2-chlorophenyl)2-(4-chlorophenyl)ethane (Pesticide)	Mice/Rat	[43,45]
DEHP (Plasticizer)	Mice/Human	[39,46]
Dieldrine (Pesticide)	Mice	[47]
Endosulfan (Pesticide)	Mice/Human	[39,48]
Fernitrothion (Pesticide)	Mice	[39]
Polycyclic aromatic hydrocarbons	Mice	[49]
Imazalil (Pesticide)	Mice	[39]
Kepone (Pesticide)	Mice	[39]
MEHP (Plasticizer)	Mice	[39]
Metolachlor (Pesticide)	Mice	[39]
Methoxychlor (Pesticide) and metabolites	Mice/Rat/Human	[39,48,50]
Monosodium methane arsenate	Mice	[39]
Nonylphenol (Plasticizer)	Human	[4]
Parathion (Pesticide)	Mice	[4]
PCB Polychlorobiphenyles (Chemical derivatives)	Mice	[45]
Propachlor (Pesticide)	Mice	[39]
2,3,7,8-Tetrachlorodibenzo-p-dioxin (TCDD)	Mice	[51]
SSS-Tributylphosphorotithioate (Pesticide)	Mice	[39]
Triclopyr (Pesticide)	Mice	[39]

**Table 3:** Drugs presenting activation or repression action on CAR.

Drugs	Species	Action	References
Neticonazole	Human	Activator	[33]
Rimcazole	Human	Activator	[33]
Sorafenib	Human	Inhibitor	[52]
Rimonabant	Human	Inhibitor	[52]
DL5050	Human	Activator	[53]
Valproic Acid	Human	Activator	[54]
Acetaminophen	Mice	Activator	[55,56]
Triazole Antifungals	Mice	Activator	[41,57]
Artemisinin	Mice/Human	Activator	[58,59]
Benzodiazepines	Human	Inhibitor	[60]
Clotrimoxazole	Human	Inhibitor	[31]
Cocaine	Human	Inhibitor	[61]
Dexamethasone	Human	Activator	[62,63]
Ketoconazole	Human	Inhibitor	[64]
Meclizine	Mice/Human	Activator (Mice) Inhibitor (Human)	[65]
Metamizole	Human	Activator	[66]
Methotrexate	Mice	Inhibitor	[67,68]
Orphenadrine	Rat	Activator	[69]
Phenobarbital	Mice, Rat	Activator	[70,71]
Phenytoin	Human	Activator	[72,73,74]
Statins	Human	Activator	[75,76]
